# Networking in microbes: conjugative elements and plasmids in the genus *Alteromonas*

**DOI:** 10.1186/s12864-016-3461-0

**Published:** 2017-01-05

**Authors:** Mario López-Pérez, Nieves Ramon-Marco, Francisco Rodriguez-Valera

**Affiliations:** Evolutionary Genomics Group, Departamento de Producción Vegetal y Microbiología, Universidad Miguel Hernández, San Juan de Alicante, Apartado 18, San Juan, 03550 Alicante, Spain

**Keywords:** *Alteromonas*, Plasmids, Pangenome, Conjugative elements, Chromid, Integrative and conjugative elements, ICE, Genomic islands

## Abstract

**Background:**

To develop evolutionary models for the free living bacterium *Alteromonas* the genome sequences of isolates of the genus have been extensively analyzed. However, the main genetic exchange drivers in these microbes, conjugative elements (CEs), have not been considered in detail thus far. In this work, CEs have been searched in several complete *Alteromonas* genomes and their sequence studied to understand their role in the evolution of this genus. Six genomes are reported here for the first time.

**Results:**

We have found nine different plasmids of sizes ranging from 85 to 600 Kb, most of them were found in a single strain. Networks of gene similarity could be established among six of the plasmids that were also connected with another cluster of plasmids found in *Shewanella* strains. The cargo genes found in these plasmids included cassettes found before in chromosome flexible genomic islands of *Alteromonas* strains. We describe also the plasmids pAMCP48-600 and pAMCP49-600, the largest found in *Alteromonas* thus far (*ca*. 600 Kb) and containing all the hallmarks to be classified as chromids. We found in them some housekeeping genes and a cluster that code for an exocellular polysaccharide. They could represent the transport vectors for the previously described replacement flexible genomic islands. Integrative and conjugative elements (ICEs) were more common than plasmids and showed similar patterns of variation with cargo genes coding for components of additive flexible genomic islands. A nearly identical ICE was found in *A. mediterranea* MED64 and *Vibrio cholera* AHV1003 isolated from a human pathogen, indicating the potential exchange of these genes across phylogenetic distances exceeding the family threshold.

**Conclusion:**

We have seen evidence of how CEs can be vectors to transfer gene cassettes acquired in the chromosomal flexible genomic islands, both of the additive and replacement kind. These CEs showed evidence of how genetic material is exchanged among members of the same species but also (albeit less frequently) across genus and family barriers. These gradients of exchange frequency are probably one of the main drivers of species origin and maintenance in prokaryotes and also provide these taxa with large genetic diversity.

**Electronic supplementary material:**

The online version of this article (doi:10.1186/s12864-016-3461-0) contains supplementary material, which is available to authorized users.

## Background

Prokaryotic microbes have sex. This has been known for very long, individuals exchange genetic material to create hybrids that have different properties from the donor cells. However, contrastingly with eukaryotes genetic exchange is independent from reproduction (cell replication) and can act on smaller sections of the genome rather than the complete hybrid generated by meiosis in eukaryotes. As a consequence, there are fewer restrains regarding donors and receptors [1]. One direct consequence of prokaryotic sex is that cells of the same species can have very different gene complements, what has led to a new paradigm, the pangenome [2], a theoretical catalog of the gene complement of the species at large. The pangenome includes a core of genes that is common to all (or most) of the strains in the taxon considered and a flexible pool that varies from strain to strain. Since the discovery and latter proposal of this term, the numbers of genes in prokaryotic pangenomes have not stopped of amazing microbiologists. A recent census in *Escherichia coli* reached close to 90,000 different gene families with 2,000 strain genomes analyzed and remarkably the discovery of new genes continue at a pace of *ca*. 300 new per strain sequenced [3]. However, in spite of the apparent enormous size of prokaryotic flexible genomes, there has to be rules that are applicable to how these genes circulate through the population.

For some years we have studied the marine bacterium *Alteromonas* with the focus on understanding its genomic make up and dynamics [4–9]. This microbe is a typical *r*-selected specialist in which success is based in high growth rates and exploitation of transient (in time or space) niches. Its species have large cells and genomes and can grow very fast when nutrients are available. The analyses of genome sequences of *Alteromonas* species isolated from all around the world revealed remarkable conservation of synteny what has allowed us to study the physical arrangement of the core and flexible regions in the genomes of representatives of this genus [9]. Using comparative genomics we proposed a model of short term evolution [9] in which clones diverge forming different clonal lineages, the smallest unit of differentiation of prokaryotes (often referred to as strains, biotypes, serotypes etc.), by acquisition of specific glycosydic receptors that were called glycotypes [9]. It has been already established that the flexible genome is largely collected in genomic islands that appear at equivalent positions in the genomes of the different isolates known as flexible genomic islands (fGIs). There are two types of fGIs with different mechanisms of variation: i) additive fGIs vary by addition or subtraction of gene cassettes, largely by site directed recombination, which movement among lineages appears to be relatively fast, and ii) replacement fGIs that code for major exposed structures of the cell (glycotype) and are exchanged by double crossover homologous recombination, using the neighboring highly conserved genes as substrate [6]. They vary at a much slower pace and remain linked for relatively longer times preventing population-wide gene sweeps [10], making a specific clone, characterized by its surface glycotype and recognized by specific populations of phages, a single selection unit [11].

To get a more complete picture of the mechanistic variation of *Alteromonas* genomes an important element was missing. What are the mechanisms by which gene pools travel from one clone to another? *Alteromonas* is not known to have natural transformation and transmission through phages, even specialized gene transfer agents, are unlikely to mediate the transfer of large genomic regions such as replacement fGIs. For these large transfers conjugation mediated by plasmids or Integrative and conjugative elements (ICEs) would be the most likely route in a Gram negative bacterium such as *Alteromonas*. Both, conjugative plasmids and ICEs (conjugative elements, CEs) have been described before in *Alteromonas* [4–6]. Plasmids are extra-chromosomal and auto-replicating DNA molecules (replicons) composed by a segment containing genetic information required for their replication and maintenance, as well as other accessory genes. Plasmid genes are normally dispensable for the essential functions of the cell. They are transferred using the *tra* system coded by themselves or by other replicons and can mobilize large segments of the chromosome that can thus be moved to different chromosomal backgrounds. ICEs are also transferable by conjugation but, unlike plasmids, they are always integrated into the host chromosome (they are not replicons per se) and have been found in both Gram-positive and Gram-negative bacteria [12]. Integration in the chromosome occurs via site-specific recombination. Although they are not as well-known as plasmids recently Guglielmini et al. [13] showed that they are the most abundant conjugative elements in practically all prokaryotic clades. These elements contain a group of core genes clustered into distinct modules required for integration/excision, conjugative transfer and regulation [14]. In addition they can acquire new DNA in specific sites proposed as hotspots by Beaber et al. [15]. Most of cargo genes (that confer adaptive functions to the host) in these modules encode for restriction modification, antibiotic and metal resistance systems [12, 16].

The aim of this study was to make a detailed comparative analysis of CEs obtained from different strains of the genus *Alteromonas* to explore the evolutionary relationships not only among the different CEs but also with the chromosomes and how they could play a role in the genomic variation (microevolution) detected at the genomic level among strains and species of the *Alteromonas* genus. The patterns found might be extrapolated at least to other aquatic Gram negative bacteria.

## Results

**Fig. 1:**
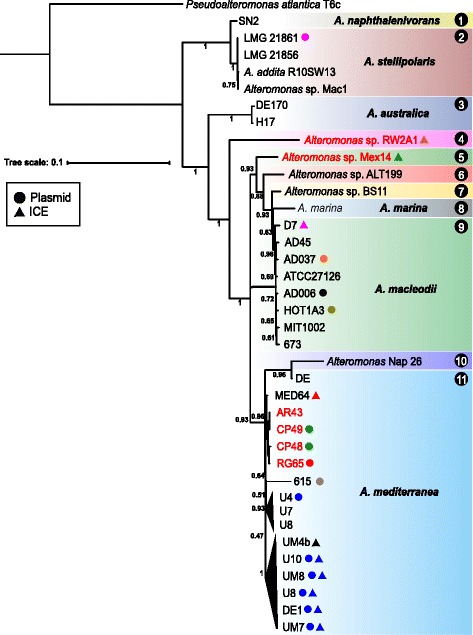
Phylogenomic tree constructed using a concatenated of the core proteome (734 proteins) in all available *Alteromonas* genomes. *Pseudoalteromonas atlantica* T6c was used as outgroup. Strains with genomes reported here for the first time are highlighted in *red*. Strains that belong to the same species are highlighted by the same colour. Presence of either plasmids or ICEs are indicated with *circles* or *triangles*, same colour indicates same version (nearly identical sequence) of the genomic element. The number in the corner of the *rectangles* indicates the number of genospecies

In order to characterize CEs we examined all the strains available in databases of the genus *Alteromonas* searching for plasmids or ICEs. In addition we are reporting here six new genomes of strains from different locations that have been fully sequenced and assembled and contain CEs (Fig. 1 and Additional file 1: Table S1). In order to investigate the phylogenomic relationships of the new *Alteromonas* strains within the *Alteromonas* genus, whole-genome phylogeny was inferred from a concatenate of the core genome (Fig. 1). Presently, strains of the genus *Alteromonas* are classified into six different species (*A. australica*, *A. marina*, *A. macleodii*, *A. mediterranea*, *A. naphthalenivorans* and *A. stellipolaris*) but using an average nucleotide identity (ANI) similarity threshold of 95% the thirty-seven strains in the tree of Fig. 1 can be classified into 11 genospecies. Our data also indicates that the new strains AR43, CP48, CP49 and RG65 are closely related and clearly belong to *A. mediterranea*. However, strains *Alteromonas* sp. Mex14 and *Alteromonas* sp. RW2A1 had similarities *ca*. 75% to the closest genospecies and could be defined as distant and novel genospecies. The general features of the new strain genomes used in this study are shown in Additional file 1: Table S1 and Fig. 1.

### Plasmids

Among the analysed *Alteromonas* strains only 14 had plasmids (Fig. 1) totalling nine different replicons (Table 1) that were found in only three different species (*A. macleodii*, *A. mediterranea* and *A. stellipolaris*). Three of them are reported here for the first time (pAMCP48-600, pAMCP49-600 and pAMRG65-300). Another plasmid (pAMBAS45) has been described in the strain *A. macleodii* AD45 [5] but we decided not to include it since it was identified as a putative defective phage. With the exception of pAMDE1-300 that was found with nearly identical sequence in different strains belonging to different *A. mediterranea* strains, and the pair pAMCP48-600/pAMCP49-600 that only differ in 7 Kb, the others were found only in one strain. Plasmid size range was from 85 Kb (pAMAD37-80) to 600 Kb (pAMCP48-600 and pAMCP49-600) (Table 1). The average GC content was always lower than the host chromosome with the highest difference (6.2%) observed for pAMRG65-300 (Table 1). Based on their mobility (presence or absence of functional conjugative machinery), plasmids can be classified in three different classes (conjugative, mobilizable and non-mobilizable). All *Alteromonas* largest plasmids >200 Kb can be classified as conjugative plasmids since they have a MOB (mobility) and MPF (membrane-associated mating pair formation) genes [17], except pASTE61-200, which has an incomplete T4SS (type IV secretion system) and thus should be classified as mobilizable (Table 1). The other three plasmids <200 Kb (pAM1A3, pAMAD6 and pAMAD37), all of them belonging to *A. macleodii* strains, lack the conjugative modules and were classified as non-mobilizable. These small plasmids probably spread by natural transformation or transduction [17].

**Table 1:** General features of the plasmids and ICEs found in *Alteromonas* genomes

CE	Size (bp)	%GC CE	%GC Chromosome	Coding Density (%)	#ORFs	#Hypothetical Protein	tRNAs	Mobility	Host	Origin	Depth(m)
pAMCP49-600	610,127	42.9	45.0	87	611	462	13	C	*A. mediterranea* CP49	Mediterranean Sea	Surface
pAMCP48-600	603,655	42.9	45.0	87	608	459	13	C	*A. mediterranea* CP48	Mediterranean Sea	Surface
pAMDE1-300	303,282	41.4	44.9	89	316	203	-	C	*A. mediterranea* DE1-UM7-U10-UM8-U4	Adriatic Sea/Ionian Sea	1,000/3,475
pAMRG65-300	302,350	38.7	44.9	86	362	100	-	C	*A. mediterranea* RG65	Mediterranean Sea	Surface
pASTE61-200	252,173	41.4	44.4	88	243	116	-	MB	*A. stellipolaris* LMG 21861	Antarctic Sea	25
pAMEC615-200	200,847	42.9	45.6	79	216	117	-	C	*A. mediterrane*a 615	English Channel	5
pAM1A3	148,934	42.3	44.8	78	209	95	-	NMB	*A. macleodii* HOT1A3	Pacific Ocean	10
pAMAD6-100	105,403	40.3	44.6	60	94	46	-	NMB	*A. macleodii* AD006	Indic Ocean	0.12
pAMAD37-85	85,188	41.1	44.7	61	89	46	-	NMB	*A. macleodii* AD037	Indic Ocean	0.12
ICE*Ama*AS1	103,910	47.9	44.9	89	98	27	-	C	*A. mediterranea* DE1-UM7-UM8-U10	Adriatic Sea/Ionian Sea	1,000/3,475
ICE*Ama*AS2	104,983	48.1	44.9	89	103	28	-	C	*A. mediterranea* UM4b	Ionian Sea	3,455
ICE*Ama*AgS1	101,205	46.0	44.8	91	98	39	-	C	*A. mediterranea* MED64	Aegean Sea	5
ICE*Ama*AnS1	117,480	47.0	44.4	88	109	32	-	C	*A. macleodii* D7	Andaman Sea	Surface
ICE*Asp*Mex1	86,518	46.0	44.2	91	73	29	-	C	*Alteromonas* sp. Mex14	Gulf of Mexico	Surface
ICE*Asp*BS1	121,528	47.3	44.4	88	112	32	-	C	*Alteromonas* sp. RW2A1	Baltic Sea	Surface

*CE* conjugative element

*C* conjugative

*MB* mobilizable

*NMB* non mobilizable

**Fig. 2:**
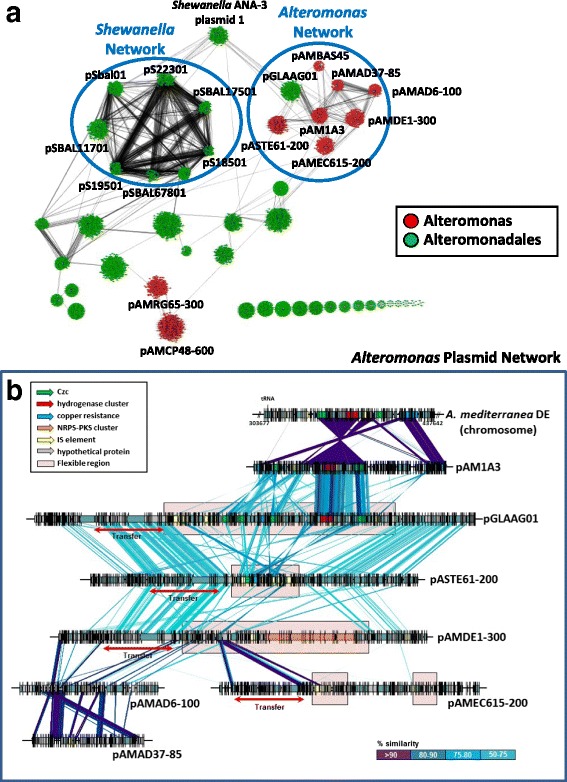
Network representing protein sharing among all the Alteromonadales plasmids. **a** A network representation of *Alteromonas* plasmids within and between all the available plasmids described at the NCBI belonging to the order Alteromonadales was produced using Cytoscape at 90% identity. *Alteromonas* plasmids are in *red*. **b** Alignment of the plasmids associated with the *Alteromonas* plasmid network

Sequence similarity (network based on 90% amino acid identities between proteins) among *Alteromonas* plasmids within and between all the populations of plasmids described at the NCBI belonging to the order Alteromonadales (46) is displayed in Fig. 2a, only 32 showed any connection. We identified two major clusters of plasmids belonging to the genera *Shewanella* and *Alteromonas*. The *Shewanella* plasmid network (SPN) is made up by eight plasmids isolated from seven *Shewanella baltica* strains and a megaplasmid (162 Kb) from *Shewanella oneidensis* MR-1 [18]. The T4SS gene cluster was their main connection (Fig. 2a). *Shewanella* ANA-3 plasmid interconnects both networks through similarity with *Glaciecola* sp. 4H-3-7 + YE-5 plasmid pGLAAG01 within the *Alteromonas* plasmid network (APN). APN contains all the *Alteromonas* plasmids except the nearly identical couple pAMCP48-600/pAMCP49-600 (only the first represented in Fig. 2) and pAMRG65-300. The last only share a few connections with the first two, all hypothetical proteins.

In order to analyze more in detail the relationships among the members of the APN we performed an alignment of the sequences (Fig. 2b). The non-mobilizable pAM1A3 [19] had a region of high similarity with pGLAAG01 that corresponds nearly exactly with a large gene cassette coding for metal resistance (clusters of *czc*ABC genes) and a hydrogenase [20] that in *A. mediterranea* DE is located in the chromosome at an additive fGI [4]. Several genomes of *Alteromonas* contain different versions of this island at the same location [7]. The presence in the plasmid of a series of gene cassettes nearly identical to some found in the *A. mediterranea* DE chromosome, indicates that plasmids are important vectors for the dispersion of these cassettes among groups of strains even beyond the genus barriers, and also how a specific combination of different operons (in this case metal resistance combined with a catabolic hydrogenase) can be acquired in a single step. The other non-mobilizable plasmids pAMAD6-100 and pAMAD37-85 showed high number of small clusters in common. These plasmids were isolated from the same sample (Port Dickson Malaysian harbor). They might share a common ancestor that lost the capacity to mobilize and since then each has diverged differentially. The two strains containing these two plasmids have an ANI of *ca*. 97%. This is consistent with them belonging to different clonal frames within the same species [21]. Interestingly, pAMDE1-300 and pASTE61-200 showed a highly syntenic region (albeit at low nucleotidic similarity from 40 to 60%) with *Glaciecola* plasmid pGLAAG01 (Fig. 2b). This region coincides with the presumed machinery for conjugal transfer and plasmid maintenance and could be designed as the plasmid “core”. All these plasmids contain also a flexible region that contains a high accumulation of IS elements that are hotspots for different gene cluster integrations (Fig. 2b). As have been previously described, in this flexible region pAMDE1-300 contained a hybrid NRPS-PKS cluster [22] and pGLAAG01 genes involved in metal resistance. However, pASTE61-200 seems that have lost not only most of the gene clusters but also some important *tra* genes suggesting that this plasmid alone cannot carry out conjugation. All this evidence illustrates how plasmids are modular, dynamic and flexible genetic scaffolds driving gene flux in their bacterial hosts over a range that might overlap at least a whole genus and beyond.

To try to detect the relationships between plasmids and chromosomes, we have investigated the presence of homologs to *Alteromonas* plasmid proteins in the genome of 225 genomes (accounting to 963,489 proteins) belonging to the eight families of the Alteromonadales order with an identity threshold set to 70% (Additional file 2: Figure S1). The results revealed that out of 5,843 plasmid proteins only 279 appeared in chromosomes at this level of similarity. Most of these homologs (38%) corresponded to the plasmid pAM1A3 and were heavy metal efflux pumps and components of the copper resistance operon [19], present in the additive fGI mentioned above. The next plasmid with more homologs (27%) was pAMCP48-600. In the latter we found that the homologs were housekeeping proteins, such as RecA, the carbon storage regulator (CsrA) or several subunits of the ribonucleotide-diphosphate reductase. Additional file 2: Figure S1 shows the abundance of plasmid similar proteins of the *Alteromonas* plasmids in all the genera within the Alteromonadales order normalized by the number of genomes within each genus. In addition, we used a 16S rRNA phylogenetic tree with 381 sequences belonging to this order to infer phylogenetic relationships (Additional file 2: Figure S1). Most of the homologs are found among closely related strains and there seems to be a negative correlation with phylogenetic distance. These results support that these plasmids have been transferred preferentially among closely related genera.

### First *Alteromonas* chromid

**Fig. 3:**
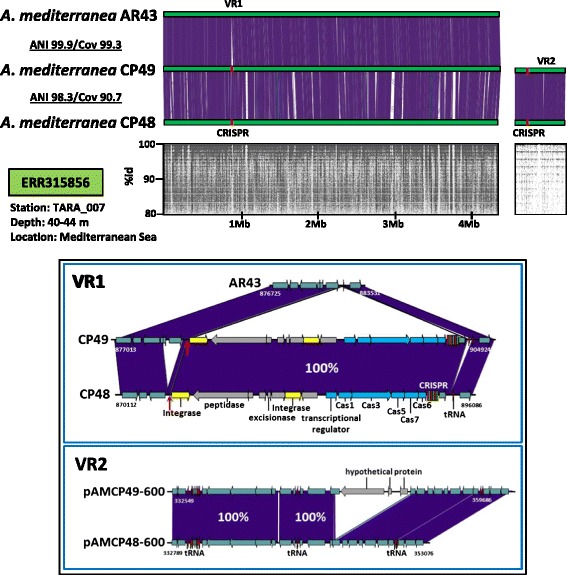
Whole-genome alignment of linearized *A. mediterranea* AR43, CP48 and CP49 genomes. The panel below indicates fragments recruited in ERR315856 TARA expedition metagenomes and their similarity to the homologous region of the CP48 chromosome and chromid. The two variables regions (VRs) are indicated. CRISPR clusters are marked in red. *Arrows* under the genome stretch represented (*lower panel*) indicate the 3’ end of the tRNA gene section that is duplicated and represents a hallmark of an integration event. Annotations of proteins in the *lower panel* are only shown for the interesting regions (CRISPR island and chromod deletion)

The megaplasmids pAMCP48-600 and pAMCP49-600 (Fig. 3) were found in two strains of *A. mediterranea* CP48 and CP49 isolated from the same place in the western Mediterranean Sea (South-eastern coast near Alicante, Spain). Although the strain chromosomes are relatively divergent (ANI 98.3%) the plasmids were nearly identical, with the main difference being a segment of 7 Kb absent in pAMCP48-600 containing three genes encoding hypothetical proteins (VR2 in Fig. 3). Interestingly, we found another strain (*A. mediterranea* AR43) isolated from a slightly different location (25 Km away) with a nearly identical chromosome to CP49 (with only 53 single nucleotide polymorphisms [SNP]) but that did not have the plasmid (Fig. 3). This indicates that this replicon is not essential for cell survival. The major difference between these strains is that AR43 has lost a small genomic island containing two gene clusters, one of them the chromosomal CRISPR/Cas system present in CP48 and CP49 (VR1 in Fig. 3). Strain CP48 although clearly belonging to a different clonal frame from CP49 (in addition to the ANI value, it has totally different versions of the four replacement fGIs described in *Alteromonas* [9]), possesses an identical CRISPR system (including the highly variable spacers) inserted in the same position (VR1 in Fig. 3). The conservation of an identical set of spacers in both CRISPR clusters is indicative of very recent common ancestry. This island (comprising the CRISPR system and another gene cluster containing a peptidase and a excisionase, Fig. 3) is located next to a single Met-tRNA that probably acted facilitating the integration of the whole island, since we found the duplicated 3’-end of the tRNA gene at the other end (red arrow in Fig. 3), a hallmark of a single integration event. In this location strains CP48 also have a small deletion of 2 Kb containing three genes coding for a restriction modification system type I (VR1 in Fig. 3). The chromosomal CRISPR system belongs to subtype I-F based on the classification made by [23], sharing identical direct repeats with another strain belonging to a different species within the genus, *A. australica* DE170, isolated from the South Adriatic Sea [7]. However, no match was obtained in the Blast searches of the 24 CP49 spacers against NCBI nr databases. Strain AR43, with a nearly identical genome to CP49 (minus the plasmid), had no trace of the island or the duplicated tRNA fragment.

**Fig. 4:**
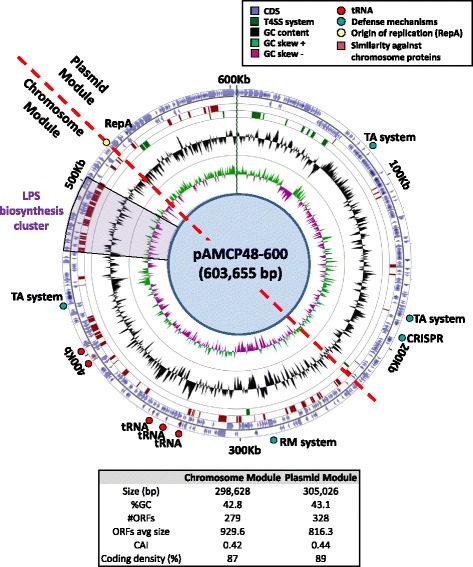
Circular representations of the pAMCP48-600 chromid. Rings from outside to inside: *Circle 1*: CDs in the positive strand. *Circle 2*: CDs in the negative strand. *Circle 3*: Stretches in red have similarity to chromosomal proteins (>50%id). *Circle 4*: In *green*, conjugation system. *Circle 5*: GC content. *Circle 6*: GC skew. *Table* indicates some general features of the two modules (chromosomal and plasmidic) that form the chromid

These megaplasmids are by far the largest *Alteromonas* plasmids published and one of the largest of all Alteromonadales and contain all the hallmarks of a “chromid” [24] including a plasmid-type replication system and paralog copies of housekeeping genes already present in the main chromosome (see below). We found 608 putative protein-coding sequences (a coding density of 87.4%) 75% of which were of unknown function. When analyzed in detail, these plasmids had actually two different segments (Fig. 4). One that retains the typical features of a plasmid, containing the *tra* gene cluster, the plasmid origin of replication and multiple hypothetical proteins we have called this half “plasmid module”. We have defined the other half as “chromosome module” (left lower side in Fig. 4). It has chromosomal features including thirteen tRNA genes that are functionally redundant, since the chromosome encoded all the tRNAs needed for protein synthesis. From the Albertsen list of orthologous markers of essential single-copy genes [25] we found five within this module in both chromids (DNA ligase, DNA polymerase III subunit beta, DNA topoisomerase IV subunit A, DNA topoisomerase IV subunit B, DNA recombination/repair protein RecA and tRNA(Ile)-lysidine synthetase). However, only one of them, coding for RecA, had significant similarity (~80%) to the chromosomal homolog. Overall, the sequence of the chromids seems to have a hybrid nature originated by the insertion of a large segment (approximately as big as the original plasmid) from a microbe distantly related to *Alteromonas* (likely another gammaproteobacterium). The insertion of the large chromosomal like segment would have occurred next to a theta origin of replication (next to the gene coding for protein RepA) (Fig. 4) [26]. We compared the position and the number of conserved or similar proteins between the chromid and chromosome by BlastP (>50%id). The result gave us a total of 83 shared proteins most of them located in the chromosomal half of the chromid (Fig. 4). Although most of them encoded proteins with unknown function there was a cluster likely involved in the biosynthesis of the lipopolysaccharide, an important component of the cell outer membrane. In order to determine the evolutionary origin of this cluster we performed several phylogenetic analyses using the amino acid sequences deduced from three essential genes (*pdaA*, *epsM* and *wzx*) implicated in the production of this polysaccharide. They all have homologs in marine microbes belonging to the Gammaproteobacteria (Additional files 3, 4 and 5: Figures S3, S4 and S5), indicating that at least this part of the chromid had been acquired from a related (albeit distantly) bacterial cell. In the plasmid module we found several genes encoding potential defense mechanisms such as a CRISPR/Cas system (different from the one in the chromosome), two toxin-antitoxin pairs and the components of the conjugative machinery (Fig. 4). However despite the differences described above, the analysis of both parts of the chromid showed no significant differences in genomic parameters such as GC content, codon adaptation index, coding density or tetranucleotide frequencies (Fig. 4).

Within the analyzed *Alteromonas* plasmids we found only two CRISPR/Cas systems, that according to the most recent classification both belong to the recently proposed type IV [23], they were found in pAMRG65-300 and the chromids (pAMCP48-600 and pAMCP49-600). This system that lacks Cas1 and Cas2 is rather rare and functionally uncharacterized. Seven and six unique spacers were identified using the CRISPR tool CRISPRFinder [27] in the repeat-spacer array preceded by three *cas* genes (*dinG*, *csf1* and *csf2*) in the plasmid and chromid respectively. We compared each CRISPR spacer sequence against the NCBI nr database as well as mobile genetic elements deposited in the ACLAME database [28] and found that only spacer 4 of the chromid repeats matched at 93% identity a putative transposase gene in the *A. macleodii* HOT1A3 plasmid (pAM1A3), this isolate was obtained from a water sample collected from the Pacific Ocean [19]. Since only a small proportion of the total diversity of microorganism is available in public databases we have analysed the presence of spacers and also the direct repeats from both CRISPR/Cas systems in the TARA metagenomes. We have found only two matches (id 100%) in all the samples and both from the same sample and same spacer (spacer 4 of the chromid). The sample corresponded to the station TARA_007 (ERR315856; Western Mediterranean Sea) the only sampling site where the chromid was detected (Fig. 3).

In order to study the global distribution of the chromid we analyzed the recruitment along the Tara Oceans transect metagenomes [29] using a cutoff at 98% nucleotide identity. pAMCP48-600/pAMCP49-600 were only detected in the station TARA_007 from the western Mediterranean Sea (Fig. 3), near the original place of isolation of the strain, indicating an endemic distribution of this conjugative element in this region of the Mediterranean and/or a recent origin. Furthermore, the host of the chromids (strains *A. mediterranea* CP48 and CP49) also recruited highly in this metagenome (Fig. 3 and Additional file 6: Table S2).

### Stability and defense mechanisms

CEs have developed systems to ensure their maintenance by postsegregational killing such as restriction-modification (RM) and toxin-antitoxin (TA) [30]. REBASE database [31] was used to identify RM genes within the plasmid sequences and their cognate chromosomes. A total of 56 RM systems (most of them belonging to the Type IV) were identified in the eight host chromosomes but only five in the plasmids. RM systems were detected in all conjugative plasmids, except pAMRG65-300, associating their presence with mobility. However, we have found some incomplete systems that have lost one of the two RM genes (the restrictase, REase or the methylase, MTase). As has been described for bacterial genomes [32] and phages [33] MTases were more abundant than REases in plasmids, suggesting a selective degradation of the REase. Similarly, TA systems are also ubiquitous and comprise bicistronic operons encoding two small genes; one gene encoding a toxic protein and the other encoding a specific antitoxin. We used the database RASTA [34] and TA finder [35] to identified potential TA systems present in both, plasmids and chromosomes. Within the chromosomes we found 94 complete TA loci belonging to the five known TA gene families (*relBE*, *HipAB*, *MazFE*, *vapBC* and *parED*). Examinations of the plasmids revealed that there is at least one plasmid-encoded TA system in each of the eight plasmids. Two TA systems were particularly prevalent in the plasmids (*relBE* and *HipAB*). Interestingly, strains belonging to *A. mediterranea* have twice the number of TA systems than the other species in the chromosome. Analysis of the location showed three hotspots of TA accumulation. Two of them corresponding to the integron and the third, that was only present in *A. mediterranea*, was located in the previously described Mobilizable Genomic Island [6]. These results indicate that TA systems are clearly associated to the flexible part of the chromosome, specifically of fGIs, the most variable regions of the genome, suggesting that they are highly mobile and widespread.

### Integrative and Conjugative Elements (ICEs)

**Fig. 5:**
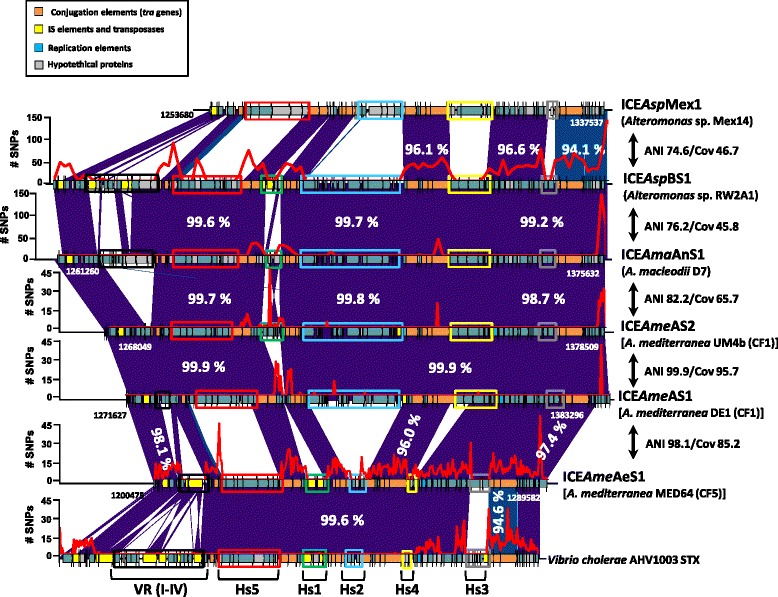
Different versions of the Integrative Conjugative Element (ICE) found in the genus *Alteromonas*. The variable region (VR) or hotspots (Hs) are highlighted by *boxes with different colors*. The number of SNPs in a 1000 bp window is indicated by a *red line*. ANI and coverage between the strains containing the ICEs is indicated to the right of the *double arrow*

ICEs are also CEs but, unlike plasmids, they are always integrated into the host chromosome. They have been found in both Gram-positive and Gram-negative bacteria [36]. We found six different ICEs in *Alteromonas* (Fig. 5), all belonging to the SXT/R391 ICE family. This family was first described in *Vibrio* and all share the same chromosomal integration site into the 5′ end of the *prf*C gene, highly conserved within species of Gammaproteobacteria [16]. The SXT/R391 ICE family has five variable hotspots (Hs). ICE*Ame*AS1 had almost identical sequence in all the isolates of clonal frame 1 of *A. mediterranea* [6]. Nearly identical ICEs were also found in two other *Alteromonas* strains belonging to two different genospecies (ANI below 95% with *A. mediterranea* or between them): *A. macleodii* D7 and *Alteromonas* sp. RW2A1, isolated from the Andaman and the Baltic Sea, they were named ICE*Ama*AnS1 and ICE*Asp*BS1 respectively (Fig. 5). Only the hotspot1 (Hs1) region was highly variable among those ICEs (Fig. 5). This Hs was variable even between two strains, *A. mediterranea* DE1 and UM4b, that belong to the same clonal frame (diverge only in 25 mutational SNPs) [9]. However, in addition to the previously described Hs in Fig. 5, some ICEs contain additional variable regions (VR) located at the left end [16]. In these regions we found in ICE*Ama*AnS1 and ICE*Asp*BS1 several different components of the *mer* operon, that confers mercury resistance to bacteria, and a calcium:sodium antiporter. *Alteromonas* sp. Mex14 and *A. mediterranea* MED64 (ICE*Asp*Mex1 and ICE*Ame*AeS1 respectively) had the most diverging ICEs (Fig. 5). However, the gene cassettes of the Hs3 region in ICE*Ame*AeS1 were identical to the same region in ICE*Ame*AS1 and the other related ICEs in the group, becoming the most conserved Hs among these ICEs.

SNP analyses showed that they were not evenly distributed throughout these ICEs and that they appeared concentrated in core genes being largely synonymous SNPs. Accumulation of large numbers of synonymous SNPs is a hallmark of high rates of homologous recombination [37]. The high similarity among the common part of the different ICEs might lead to frequent recombination and replacement by double crossover of the Hs genes (Fig. 5). On the other hand, the low amounts of SNPs in the Hs genes indicates that they are very rapidly exchanged preventing synonymous SNPs to accumulate, as has been proposed for additive fGIs in the chromosome [9]. By contrast, the variability found in the VRs is likely mechanistically simpler, using IS elements as drivers of the diversity. Genes in these modules are designated cargo genes. Most of cargo genes in *Alteromonas* encode restriction modification or metal resistance systems and can confer diverse capabilities to the organisms that contain the ICE. Contrastingly, in the pathogenic vibrio strains, cargo genes were mostly involved in transport and antibiotic resistance, probably critical for their clinical relevance.

**Fig. 6:**
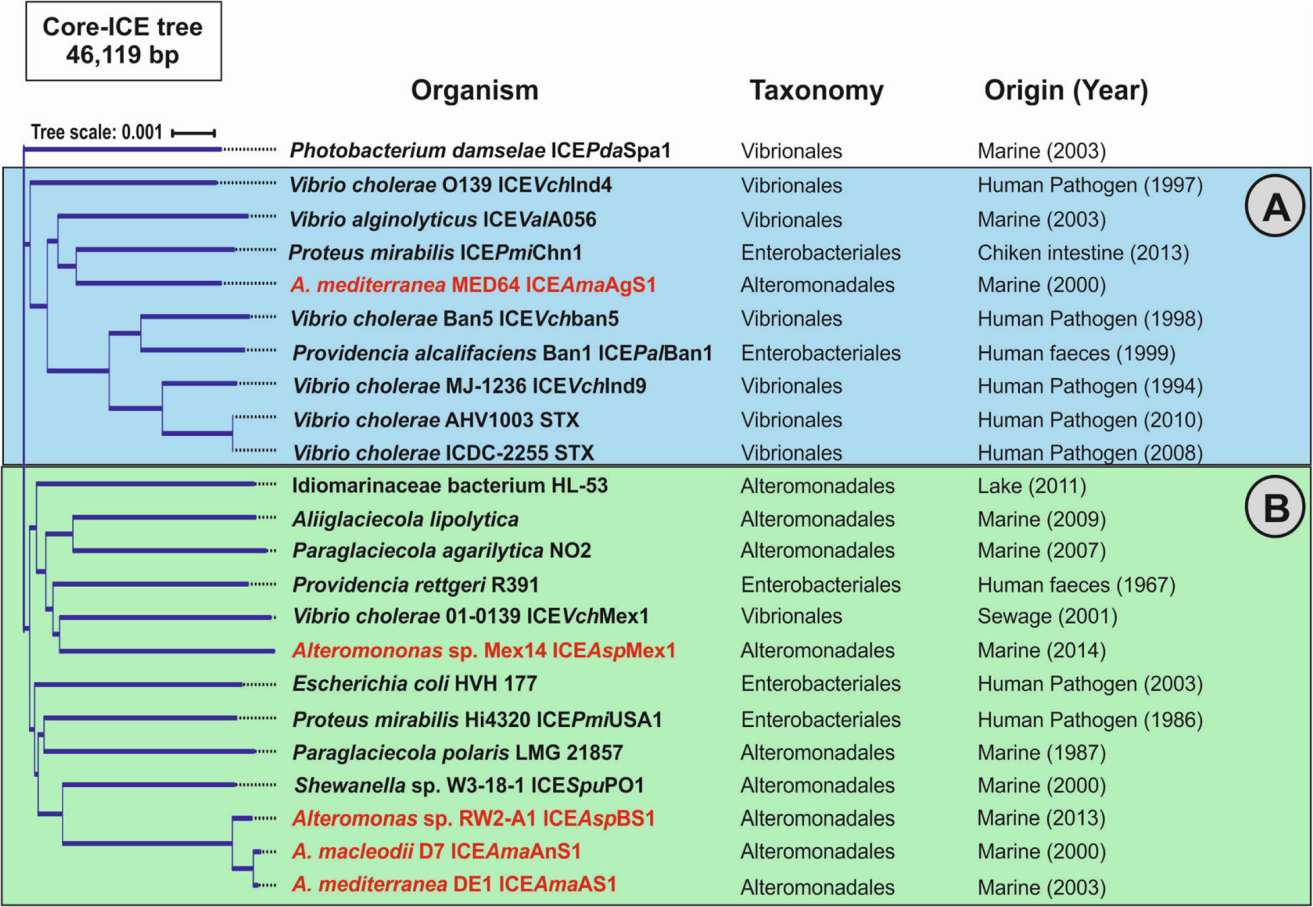
Phylogenetic tree constructed using a concatenate of the core (shared genes) in all ICEs of the family SXT/R391 available. The two clusters designated **a** and **b** are highlighted in different *colour. Photobacterium damselae* ICE*Pda*Spa1 appears as an outgroup. *Alteromonas* ICEs are highlighted in *red*

To asses and compare the variability within the SXT/R391 family we used the six different ICEs found in *Alteromonas* together with all the complete SXT/R391 ICEs available in the databases [38] comprising both environmental and clinical isolates of several Gammaproteobacteria species (Fig. 6). Moreover, using ICE*Ame*AS1 as reference we were able to assemble another three new ICEs from the contigs of three marine members belonging to the recently reclassified *Glaciecola* strains (*Aliiglaciecola lipolytica*, *Paraglaciecola agarilytica* NO2 and *Paraglaciecola polaris* LMG21857). Synteny of the SXT/R391 ICEs was well preserved having sizes ranging from 74 to 121 Kb. Whole genome alignment among the 23 SXT/R391 ICEs revealed a total of 15 homologous regions representing a core of 46 Kb, containing 48 genes, with 6399 variables sites and a 96.5% average identity at the nucleotide level. In order to analyse the evolution of the SXT/R391 family we created a phylogenetic tree using the concatenated core (Fig. 6). The resulting tree showed that, leaving aside *Photobacterium damselae* ICE*Pda*Spa1, there are two clusters that we designated A and B (Fig. 6). Although most of the ICEs belonging to *Alteromonas* species, together with other marine microbes, fell into group B, ICE*Ama*AgS1 (*A. mediterranea* MED64) was grouped with most of the pathogenic vibrios. In addition, three pathogenic Enterobacteriaceae (*Providencia rettgeri* R391*, Proteus mirabilis* Hi43320 and *Escherichia coli* HVH177) showed close relationship with members of group B suggesting that saprophytic and free living microbes share the same pool of ICEs. Interestingly, in spite of the different locations and environmental origins (marine and human patient), the ICE sequences of *A. mediterranea* MED64 ICE*Ame*AeS1 and *V. cholera* AHV1003 were found to be almost identical (58 Kb with 99.6% of identity). The only difference at the level of hotspots was that both had different gene cassettes at Hs3 (Fig. 5). In addition, AHV1003 carried many antibiotic resistance genes in the four variable regions (VR I-IV) that conferred resistance to tetracycline and erythromycin, critical to the clinical relevance of this strain. We also performed pairwise BLAST comparison with all the ICEs showing that nearly identical cassettes were frequently detected regardless of the origin of the strain (clinical or environmental) and disconnected from the core degree of relatedness (data not shown).

## Discussion

We have studied genomes of *Alteromonas* as a model to understand the evolutionary dynamics of a marine free-living bacterium [9]. The fact that *Alteromonas* is not known to associate to humans or marine animals simplify the interpretation and can shed light on other marine free-living microbes. It can also help understanding the ones that are transient human pathogens such as *V. cholera* [39]. Here we have focused on the plasmids and ICEs that can provide the fastest way to mobilize genomic regions to create new combinations and expand the physio-ecological range of the microbe. We have purposefully excluded lysogenic phages because, although they can transfer host DNA, it is much more limited quantitatively and has other major role as a strategy of virus survival that is hard to discriminate from their role in host evolution. Gene transfer agents [40] have been also obviated, although they could be carriers of large genomic fragments, but the comparative genomics approach used here would not be able to detect them. We have found a relatively small number of plasmid and ICEs, only 24 among 37 strain genomes. However, their role could be paramount in the networks of genomic communication among these microbes if they flow fast throughout the population. Although the plasmids described here seemed to be restricted to a single species, pAMADE1-300 was found in different strains of *A. mediterranea* pointing to a fast dispersion rate. In addition, a clear connection was found between the plasmids of *Alteromonas* and *Glaciecola*, indicating networks of these elements that extend beyond a single genus. Actually, the identification of similar genes in isolates belonging to even different families (Vibrionaceae) attests to a broad range of hosts at least of the ICEs described here. Although the core functions of the ICEs such as the mechanisms to promote their integration, excision, transfer and regulation have been well studied [41] and mainly have been characterized as key vectors of antibiotic resistance, little is known about the evolutionary dynamics of these elements in aquatic environments. Our comparative analyses showed the importance of inter-element recombination producing hybrid ICEs. The dynamics of variation of these elements based on the continuous change of the different modules recruited from an extensive pool, likely distant *Gammaproteobacteria*, is fast enough to mask evolutionary history.

We have found examples that could illustrate how the two major kinds of fGIs i.e. additive and replacement [6, 9] can travel among relatively distant strains. The case of the large hydrogenase-metal resistance gene cluster [20] sharing nearly identical sequences in a conjugative plasmid and in the chromosome of different species of *Alteromonas* is a paradigmatic example of how an additive fGI can be exchanged. In the case of replacement fGIs [6] the evidence is less compelling but the polysaccharide cluster found in chromids pAMCP48-600/pAMCP49-600 could reflect an intermediate step in the process of exchange of a replacement fGI. The chromid would provide an alternative gene cluster that eventually could replace the chromosomal one. However, the lack of a similar cluster found in any *Alteromonas* strain or other related microbe prevents concluding that this would be a viable path for this kind of fGI to be exchanged. We have previously formulated a theory by which replacement fGIs flow much more slowly throughout the clonal frames that constitute a species, allowing time for them to diverge at the level of microniche specialization [9]. Along these lines, it is not surprising that, with the relatively small numbers of strains analysed, no “smoking gun”, that is a replacement fGI caught in the process of being exchanged, would surface.

Regarding the finding of the strains (CP48 and CP49) containing the same chromid and CRISPR/Cas system connected to two clearly divergent chromosomes, the most parsimonious explanation is that CP48 has received both the plasmid and the CRISPR cassette from a relative of CP49. In both cases the CP48 fragments have small deletions that might have happened more easily than insertions in CP49 (Fig. 3). In addition, AR43 has a little insertion at the left hand side of the island. Exchange of the chromid and the CRISPR/Cas system between strains CP48 and CP49 must have happened in a short timeframe given the nearly identical sequence of the chromids and the identical spacers shared by the CRISPRs. Although the plasmid was detected only in two strains isolated simultaneously, its recruitment from a distant location (Fig. 3) illustrated that it can remain as a stable entity for a significant amount of time. Its presence in two widely divergent genomic backgrounds (strains with only 98% ANI) illustrates how it can move fast in the population of divergent *A. mediterranea* clones, in spite of a significant decrease in growth rate (data not shown) induced by the presence of the chromid.

## Conclusion

Findings presented here underscore the relevance of CEs as main vectors in the exchange of gene cassettes in the chromosomal additive fGIs. We have found the first chromid described in *Alteromonas* containing two clearly distinguishable parts designed as “chromosomal” and “plasmid” modules. The chromid contained a LPS biosynthesis cluster that provides a clue as to how replacement fGIs might be exchanged among clonal lineages. Our analyses also revealed that the continuous change of the different modules by recombination is an important mechanism to produce ICE variation and occur even at phylogenetic distances exceeding the family threshold, since we have found nearly identical ICEs in *A mediterranea* MED64 and *V. cholera* AHV1003, a human pathogen.

## Methods

### Sample collection, sequencing, assembly and annotation

Details of isolation and origin of the new *Alteromonas* strains using are provided in Additional file 1: Table S1. Strains AR43, CP48, CP49 and RG65 were isolated from a Mediterranean seawater sample collected off the coast of Alicante (Spain). Strain Mex14 was isolated from a sample obtained in an aquaculture pond filled up with water from the Gulf of Mexico located in Sisal (Yucatan). RW2A1 came from a coastal seawater sample from the Baltic Sea. DNA was extracted by phenol-chloroform as described in [42] and checked for quality on a 1% agarose gel. The quantity was measured using Quant-iT® PicoGreen ® dsDNA Reagent (Invitrogen). Genomic DNA (5 μg) was sequenced using an Illumina HiSeq 2500 platform with 100 bp paired end reads at BGI Tech Solutions (Hong Kong). The trimmed sequences were assembled de novo using IDBA 1.1.1 [43] and Geneious (http://www.geneious.com/). PCR primers were designed from the sequence of the ends of assembled contigs to obtain single closed contigs. Genomes were annotated using the NCBI PGAAP annotation pipeline (http://www.ncbi.nlm.nih.gov/genome/annotation_prok/). The predicted protein sequences were compared using BLASTP to the NCBI nr protein database (e-value 10–5). ORFs smaller than 100 bp and without significant homology to other proteins were not considered. Reciprocal BLASTN (>50% query coverage and >90% identity) and TBLASTXs (>50% query coverage and >50% identity) searches between the genomes were carried out, leading to the identification of regions of similarity, insertions and rearrangements. The average nucleotide identity (ANI) between strains was calculated using JSpecies software package v1.2.1 using default parameters [44]. To allow the interactive visualization of genomic fragment comparisons Artemis v.12 [45], Artemis Comparison Tool ACTv.9 [46] were used to compare genomes. Additional local BLAST searches against the latest NCBI nr database (September 2016) were performed whenever necessary. CRISPRFinder was used to screen for the presence of CRISPR arrays [27].

### CEs analysis

All the available complete plasmid sequences belonging to the order Alteromonadales were downloaded from the NCBI (September 2016). To determine the relationship among the plasmids in terms of shared proteins, a similarity plasmid network was constructed with Cytoscape [47] using the protein sequence data set in an all versus all BLAST [48]. We considered only significant an amino acid identity of 90% and the alignment covering not less than 70% of the query sequence. All the complete ICEs belonging to the family SXT/R391 were obtained from the web-based resource ICEberg [38]. Multiple alignment of genomic sequences for all the ICEs was performed by using Mauve multiple alignment software (v2.3.1) obtaining genomic regions shared among all of them with a minimum length of 500 bp [49]. The resulting alignments were subsequently used in ClonalFrame software v1.2 using default parameters [50]. The clonal genealogy inferred from the generated alignment of 46 Kb of the core genome by ClonalFrame is shown in Fig. 6. Nucmer program in the MUMmer3+ package [51] was used to identify the indels and the SNPs between small regions of the ICEs using default parameters. The total number of SNPs ICEs was calculated for each pair of to obtain the average in a 1000 bp window.

### Phylogenetic analysis

To determine the exact phylogenetic relationship of the new isolates within the genus, phylogenomic analysis for all the *Alteromonas* members whose genomes were available were carried out (Fig. 1). The phylogenomic tree was rooted using *Pseudoalteromonas atlantica* T6c (NC_008228.1) as an outgroup. The complete genomes were analyzed using TIGRfams to identify all the conserved proteins (734). The concatenated proteins were aligned using Kalign [52] and a maximum likelihood tree was made using FastTree [53] using a JTT + CAT model and a gamma approximation. Interactive Tree Of Life (http://itol.embl.de), a web-based tool, was used to display and manipulate the tree. The 16S rRNA gene and the proteins involved in the biosynthesis of the lipopolysaccharide were aligned with clustalW and the evolutionary phylogenetic trees were constructed using neighbour-joining calculated using Kimura’s two-parameter model, with the robustness of 100 replications, using MEGA 5 software [54]. Most similar Lipopolysaccharide biosynthesis protein sequences were recovered from NCBI (all sequences) and for the 16S rRNA gene tree only the sequences belonging to validly named genus of the order Alteromonadales were used.

### Recruitments of environmental collections

Genomes and CEs recruitments were carried out against the complete data set of Tara Ocean metagenomes [29]. BLASTN was carried out, and a very restrictive cut-off of 99% identity in 70% of the length of the environmental read was established to guarantee that only similarities at the level of nearly identical microbes were included. However, the cut-off for the chromid recruitment was established at 95% of identity. These hits were used to compute the RPKG (reads recruited per Kb of genome per Gb of metagenome) values that provide a normalized number comparable across various metagenomes.

### Databases

ACLAME [28]: a collection and classification of prokaryotic mobile genetic elements; ICEberg [38]: a web-based resource for integrative and conjugative elements found in Bacteria; RASTA [34]: a web-based tool for identifying toxin-antitoxin loci in prokaryotes; REBASE [31]: a database for DNA restriction and modification; TA finder [35]: a web-based tool to identify Type II toxin-antitoxin loci in bacterial genome.

### Accession numbers

The genome and CE sequences have been deposited in GenBank under the following BioProject accession number PRJNA352689.

## Additional files

Additional file 1: Table S1. General features of *Alteromonas* genomes used in this study. (PDF 2353 kb)
Additional file 2: Figure S1. Number of proteins of *Alteromonas* plasmids with similar homologs in the genome of 225 strains belonging to the eight families of the Alteromonadales. The numbers of genomes for each genus are indicated between brackets after each genus name. The total numbers of predicted proteins in all the genomes that match proteins in the plasmids are indicated at the bottom. The colour coded heat map (1–5) measures the relative abundance of hits for each plasmid normalized by the total number of Mb of genomes of the corresponding genus used in the comparison. A 16S rRNA tree is shown on the left to give a framework of phylogenomic distance to *Alteromonas*. (PDF 2353 kb)
Additional file 3: Figure S2. Phylogenetic relationships of lipopolysaccharide biosynthesis genes in the chromid. Tree based on 82 lipopolysaccharide biosynthesis protein (*wzx*) alignment. Protein sequences were aligned with clustalW and phylogenetic tree was constructed using a neighbour-joining model with 100 replications. (PDF 2353 kb)
Additional file 4: Figure S3. Phylogenetic relationships of lipopolysaccharide biosynthesis genes in the chromid. Tree based on 94 exopolysaccharide biosynthesis protein (*eps*M) alignment. Protein sequences were aligned with clustalW and phylogenetic tree was constructed using a neighbour-joining model with 100 replications. (PDF 2353 kb)
Additional file 5: Figure S4. Phylogenetic relationships of lipopolysaccharide biosynthesis genes in the chromid. Tree based on 104 polysaccharide deacetylase (*pda*A) protein alignment. Protein sequences were aligned with clustalW and phylogenetic tree was constructed using a neighbour-joining model with 100 replications. (PDF 2353 kb)
Additional file 6: Table S2. Recruitment values in TARA Ocean expedition metagenomes for the chromids (pAMCP48-600/pAMCP49-600) and the chromosome of strains (CP48 and CP49) where the RPKG values (reads per kilobase per gigabase of data) were significant (>5RPKG). (PDF 2353 kb)

## Abbreviations

ANI: Average nucleotide identity
APN: *Alteromonas* plasmid network
CEs: Conjugative elements
fGIs: flexible genomic islands
Hs: Hot spots
ICEs: Integrative and conjugative elements
MTase: Methyltransferase
REase: Restriction endonuclease
RM: Restriction-modification system
RPKG: Reads recruited per Kb of genome per Gb of metagenome
SNP: Single nucleotide polymorphisms
SPN: *Shewanella* plasmid network
T4SS: Type IV secretion system
TA: Toxin-antitoxin
VR: Variable region
