# Supplementary material for: Networking in microbes: conjugative elements and plasmids in the genus Alteromonas
Source: BMC Genomics. 2017 Jan 5;18:36. doi: 10.1186/s12864-016-3461-0 (PMC5217437; doi:10.1186/s12864-016-3461-0)
Supplement: Additional file 1: Table S1. — General features of Alteromonas genomes used in this study. (PDF 2353 kb) [file 12864_2016_3461_MOESM1_ESM.pdf]

Additional file 1: Table S1. General features of *Alteromonas* genomes used in this study.

Strain	Size (bp)	%GC	ORFs	Plasmid	ICE	Date	Origin	Depth(m)	Reference
A. mediterranea **615**	4,590,508	45.6	4,546	pAMEC615-200	-	2005	English Channel	5	Southward et al, 2005
A. mediterranea **DE1**	4,947,126	44.9	4,347	pAMDE1-300	ICE*Ama*AS1	2003	Adriatic Sea	1,000	López-López et al, 2005
A. mediterranea **UM4b**	4,438,767	44.9	3,898	-	ICE*Ama*AS2	1998	Ionian Sea	3,455	Sass et al, 2001
A. mediterranea **MED64**	4,397,537	44.8	4,050	-	ICE*Ama*AgS1	2000	Aegean Sea of Lebanon	5	Pinhassi and Berman, 2003
A. mediterranea **CP48**	4,928,072	45.0	3,602	pAMCP48-600	-	2014	Mediterranean Sea	Surface	This work
A. mediterranea **CP49**	4,964,463	45.0	3,662	pAMCP48-600	-	2014	Mediterranean Sea	Surface	This work
A. mediterranea **AR43**	4,344,564	45.0	3,647	-	-	2014	Mediterranean Sea	Surface	This work
A. mediterranea **RG65**	4,652,339	44.9	3,640	pAMRG65-300	-	2014	Mediterranean Sea	Surface	This work
A. macleodii **HOT1A3**	4,801,807	44.8	3,949	pAM1A3	-	2007	Pacific Ocean	10	Sher et al, 2011
A. macleodii **AD006**	4,728,413	44.6	3,975	pAMAD6-100	-	2014	Indic Ocean	0.12	This work
A. macleodii **AD037**	4,703,088	44.7	3,991	pAMAD37-85	-	2014	Indic Ocean	0.12	This work
A. stellipolaris **LMG 21861**	4,904,192	44.4	4,078	pASTE61-200	-	1999	Antarctic Sea	25	Van Trappen et al, 2004
A. macleodii **D7**	4,575,620	44.4	3,833	-	ICE*Ama*AnS1	2000	Andaman Sea	Surface	This work
Alteromonas **sp. Mex14**	4,489,455	44.2	3,794	-	ICE*Asp*Mex1	2014	Gulf of Mexico	Surface	This work
Alteromonas **sp. RW2A1**	4,107,146	44.4	3,628	-	ICE*Asp*BS1	2013	Baltic Sea	Surface	This work
