# Supplementary material for: Networking in microbes: conjugative elements and plasmids in the genus Alteromonas
Source: BMC Genomics. 2017 Jan 5;18:36. doi: 10.1186/s12864-016-3461-0 (PMC5217437; doi:10.1186/s12864-016-3461-0)
Supplement: Additional file 2: Figure S1. — Number of proteins of Alteromonas plasmids with similar homologs in the genome of 225 strains belonging to the eight families of the Alteromonadales. The numbers of genomes for each genus are indicated between brackets after each genus name. The total numbers of predicted proteins in all the genomes that match proteins in the plasmids are indicated at the bottom. The colour coded heat map (1–5) measures the relative abundance of hits for each plasmid normalized by the total number of Mb of genomes of the corresponding genus used in the comparison. A 16S rRNA tree is shown on the left to give a framework of phylogenomic distance to Alteromonas. (PDF 2353 kb) [file 12864_2016_3461_MOESM2_ESM.pdf]

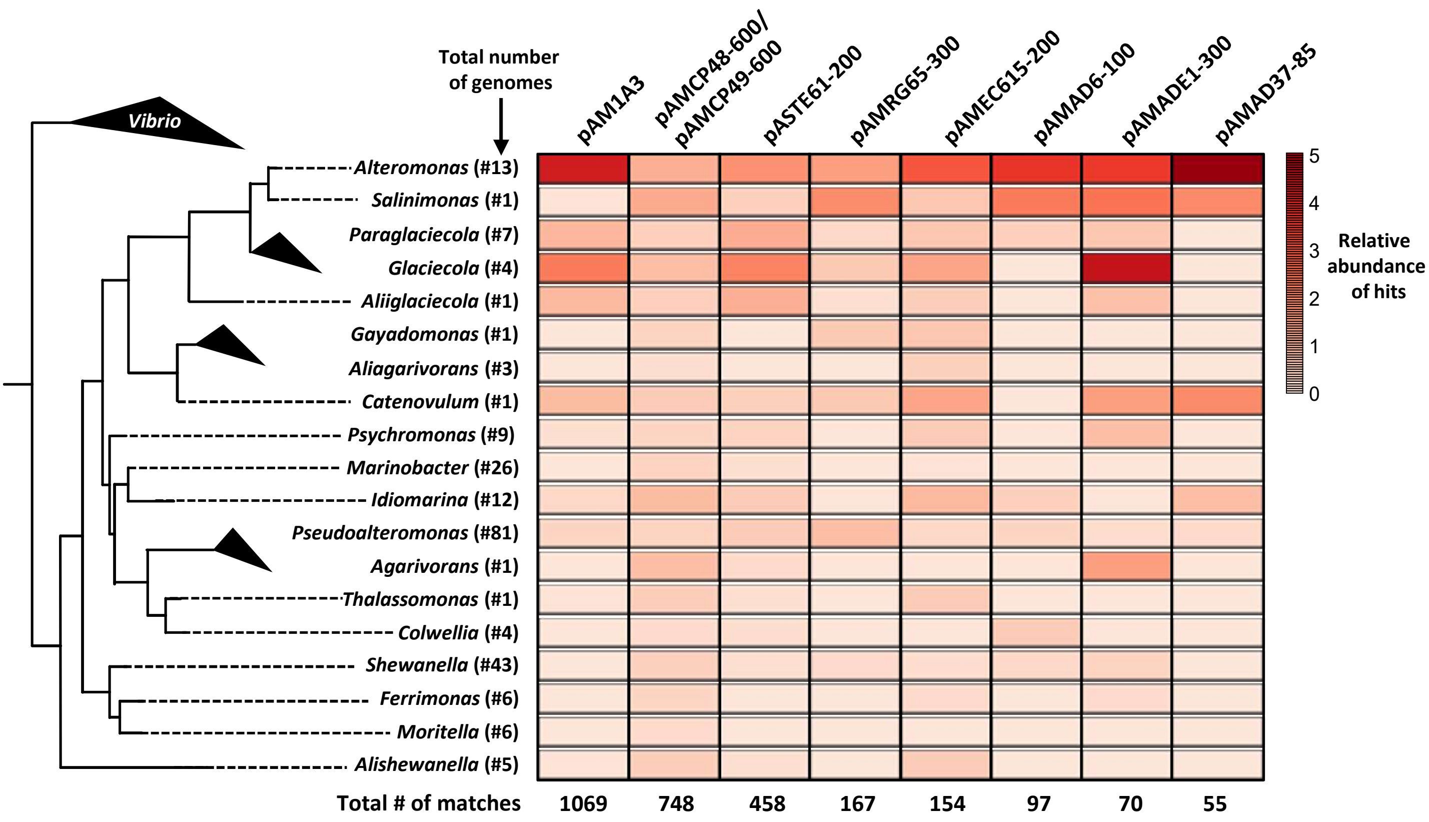

Additional file 2: Figure S1. Number of proteins of *Alteromonas* plasmids with similar homologs in the genome of 225 strains belonging to the eight families of the Alteromonadales. The numbers of genomes for each genus are indicated between brackets after each genus name. The total numbers of predicted proteins in all the genomes that match proteins in the plasmids are indicated at the bottom. The color coded heat map (1-5) measures the relative abundance of hits for each plasmid normalized by the total number of Mb of genomes of the corresponding genus used in the comparison. A 16S rRNA tree is shown on the left to give a framework of phylogenomic distance to *Alteromonas*.
