# Supplementary material for: Networking in microbes: conjugative elements and plasmids in the genus Alteromonas
Source: BMC Genomics. 2017 Jan 5;18:36. doi: 10.1186/s12864-016-3461-0 (PMC5217437; doi:10.1186/s12864-016-3461-0)
Supplement: Additional file 5: Figure S4. — Phylogenetic relationships of lipopolysaccharide biosynthesis genes in the chromid. Tree based on 104 polysaccharide deacetylase (pdaA) protein alignment. Protein sequences were aligned with clustalW and phylogenetic tree was constructed using a neighbour-joining model with 100 replications. (PDF 2353 kb) [file 12864_2016_3461_MOESM5_ESM.pdf]

Polysaccharide deacetylase protein (PdaA)

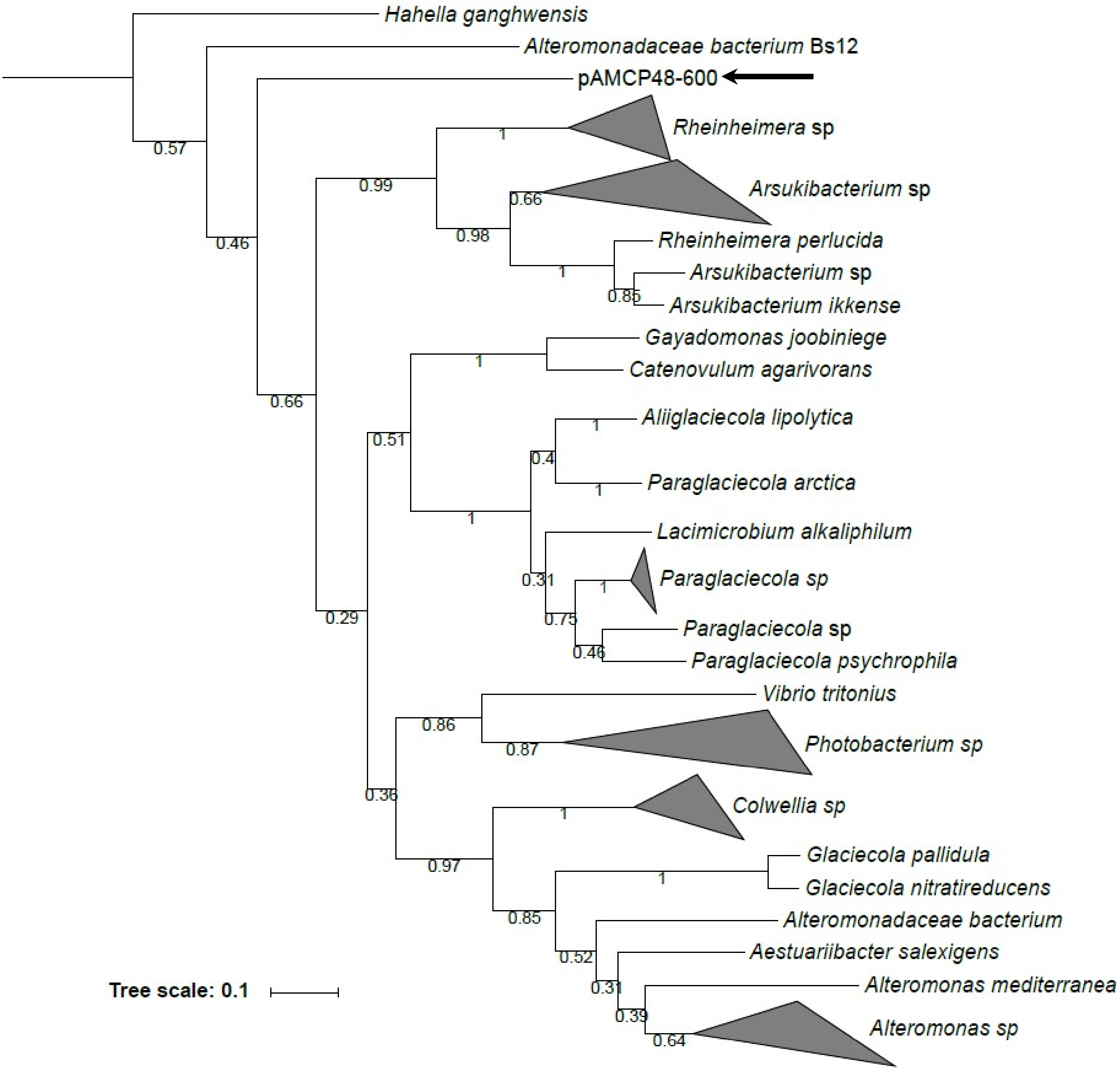

Additional file 5: Figure S4. Phylogenetic relationships of lipopolysaccharide biosynthesis genes in the chromid. Tree based on 104 polysaccharide deacetylase (*pda*A) protein alignment. Protein sequences were aligned with clustalW and phylogenetic tree was constructed using a neighbour-joining model with 100 replications.
