# Supplementary material for: Networking in microbes: conjugative elements and plasmids in the genus Alteromonas
Source: BMC Genomics. 2017 Jan 5;18:36. doi: 10.1186/s12864-016-3461-0 (PMC5217437; doi:10.1186/s12864-016-3461-0)
Supplement: Additional file 6: Table S2. — Recruitment values in TARA Ocean expedition metagenomes for the chromids (pAMCP48-600/pAMCP49-600) and the chromosome of strains (CP48 and CP49) where the RPKG values (reads per kilobase per gigabase of data) were significant (>5RPKG). (PDF 2353 kb) [file 12864_2016_3461_MOESM6_ESM.pdf]

Additional file 6: Table S2. Recruitment values in TARA Ocean expedition metagenomes for the chromids (pAMCP48-600/pAMCP49-600) and the chromosome of strains (CP48 and CP49) where the RPKG values (reads per kilobase per gigabase of data) were significant (>5RPKG).

#SEQID	ERR315857	ERR315856	ERR594405	ERR594323
A. mediterranea CP48	3.64	85.66	24.69	7.62
A. mediterranea CP49	3.54	86.67	24.69	7.64
pAMCP48-600/pAMCP49-600	0.01	7.17	0.06	0.02
Metagenome Location	Mediterranean Sea	Mediterranean Sea	Mediterranean Sea (East)	South Pacífic Ocean
Filter Size	0.22mm	0.22mm	< 0.22mm	0.45mm
Depth	5m	42m	70m	5m

#SEQID	ERR594284	ERR594301	ERR594319	ERR594309
A. mediterranea CP48	12.15	5.93	18.54	4.89
A. mediterranea CP49	11.99	5.83	18.10	4.73
pAMCP48-600/pAMCP49-600	0.05	0.01	0.09	0.04
Metagenome Location	South Pacífic Ocean	South Pacífic Ocean	South Pacífic Ocean	South Pacífic Ocean
Filter Size	0.22mm	0.45mm	0.45mm	0.22mm
Depth	115m	115m	150m	600m

#SEQID	ERR594322	ERR594333	ERR594289
A. mediterranea CP48	3.21	7.99	17.47
A. mediterranea CP49	3.15	8.08	17.29
pAMCP48-600/pAMCP49-600	0.04	0.08	0.14
Metagenome Location	South Pacífic Ocean	South Atlantic Ocean(West)	South Atlantic Ocean(West)
Filter Size	0.45mm	0.45mm	0.45mm
Depth	600m	800m	800m

#SEQID	ERR594299	ERR599149	ERR594331
A. mediterranea CP48	3.32	9.35	26.01
A. mediterranea CP49	3.23	8.91	26.09
pAMCP48-600/pAMCP49-600	0.01	0.02	0.12
Metagenome Location	South Atlantic Ocean(East)	South Atlantic Ocean(East)	South Atlantic Ocean(East)
Filter Size	0.22mm	0.22mm - 3 mm	0.45mm
Depth	800m	800m	800m

#SEQID	ERR594295	ERR594327	ERR594312	ERR594290
A. mediterranea CP48	154.73	8.11	10.39	13.07
A. mediterranea CP49	155.18	7.96	10.16	12.91
pAMCP48-600/pAMCP49-600	0.02	0.03	0.03	0.10
Metagenome Location	Arabian Sea	Arabian Sea(South)	Arabian Sea(North)	Arabian Sea(North)
Filter Size	0.22mm	0.22mm	0.22mm	0.22mm
Depth	5m	5m	340m	600m
